# Water contact angle is not a good predictor of biological responses to materials

**DOI:** 10.1116/1.4989843

**Published:** 2017-07-06

**Authors:** Morgan R. Alexander, Paul Williams

**Affiliations:** School of Pharmacy, University of Nottingham, Nottingham, United Kingdom; School of Life Sciences, University of Nottingham, Nottingham, United Kingdom

## Abstract

Often the view is expressed that water contact angle (WCA) or other wettability/surface energy measurements made on a material surface can be used to predict cellular attachment to materials, e.g., *bacteria attach to hydrophobic surfaces.* In this article, the authors present a perspective emerging from their work that has failed to find relationships between WCA and microbial and stem cell attachment within large diversity material libraries and compare with the literature concluding that such simple rules are (unfortunately) wholly inadequate to explain cell–material interactions.

## I Introduction

Although it is clear to many that the water contact angle (WCA) measured on a material surface is not a good general predictor of cellular response to that surface, the concept of *hydrophobic* or *hydrophilic* surfaces having certain cellular attachment properties is often encountered. In this letter, we outline why we believe that this misconception arises.

Our view is that the source of confusion is an over simplification and misunderstanding of careful studies of biological interactions with model surfaces and the high profile of hydrophilic attachment resistant materials such as ethylene glycols and hydrogels. We believe that studies on model surfaces are being incorrectly extrapolated to material surfaces in general, where many possible differences between the surfaces prevent a measure as simple as wettability from having any predictive capacity. This observation is not new, the authors who carried out the original studies often noted this and included these caveats in their work and a number of others have picked up this theme. We revisit it here because of the new data from our recent high throughput works gathered from polymer microarray libraries.

We hope that the discussion we aim to stimulate is in the spirit of the celebration of the career of Buddy Ratner on his 70th birthday held at PacSurf, where Morgan Alexander presented a talk containing a slide on this subject. Buddy’s career coincided with the transition of analytical biomaterial science from being dependent on surface energetics and solution assay measurements of surface chemistry, to the current era where researchers have available a plethora of spectroscopic analytical surface science techniques to provide quantitative measures of surface elemental, function, and molecular structure, many pioneered by him! We do not present this as a full review but rather a *letter to the editor* to contextualize our recent polymer library observations in the wider literature of WCA and surface energetics. For an indepth review of the area of biomaterials and surfaces, the reader is directed to articles by Ratner and others.^1–5,39–41^

## II Motivation

The interface between cells and man-made materials is of importance in many fields, ranging from microbial colonization of, and biofilm development on, maritime structures, host tissues, and implanted medical devices to immune system rejection of such implants as well as plastic culture-ware substrates for the *in vitro* support and expansion of mammalian cells. The material–cell interface is also key for controlling the body’s interactions with implants, from biosensors to emerging regenerative medicine scaffolds that support tissue regeneration. Consequently, the importance of cell attachment to man-made materials impacts on the environment in the form of inefficient fuel usage caused by fouled maritime surfaces and human mortality and morbidity associated with the failure of medical devices. As clearly evidenced by Ratner and others, *in vitro* biological performance involving protein adsorption and mammalian cell growth is directly related to the surface properties of these materials, including chemistry, topography, and stiffness.^1^ While in many cases, it is the *in vivo* performance that is ultimately of interest, we will restrict this article to considering the more controllable *in vitro* challenge environments. We will also focus primarily on the effects of *non-specific* material chemistry interactions at the surface, i.e., not utilizing surface bound biological epitopes. This focus is taken with the understanding that in most culture environments, the influence material surface chemistry has on attached cells is mediated by adsorption of biomolecules. The importance of surface compliance^6^ and topography^7,8^ is also well studied and identified in certain cell–surface systems where these factors can be controlled, while the chemistry is kept constant-this is beyond the scope of this letter.

When presenting structure-property results from large polymer libraries at scientific conferenes noting the correlation of bacterial biofilm formation with polymer chemistry, we have frequently been asked about the relatively *hydrophobic* nature of the surface of the materials that we found to resist bacterial attachment, given that *hydrophilic materials* are usually thought to be best for resisting bacterial adhesion. This reveals a widely held impression that “hydrophilic materials” resist bacterial attachment. We have been taught to describe things in the simplest manner possible, employing Occam’s razor. Using the simple parameter of water contact angle (WCA)/wettability is therefore attractive, even to explain such a complex phenomenon as cellular responses to man-made surfaces in culture media or simulated medical device service environments. We argue that to think of the performance in terms of surface wettability alone is not helpful. We also contend that converting this into surface energy after measuring multiple contact angles from liquids of different surface tension does not greatly improve the situation.

## III Background of Wettability Measurements

A sessile drop water contact angle measurement is likely the first surface measurement made by prehistoric mankind, when visual observation of the beading of rain droplets marked out the waxy hydrophobic leaves of certain plants as good barriers to wet weather. Subsequently, wettability would have been a good measure of how weather resistance properties degrade upon ageing of the leaves—possibly the first observation of a surface structure-property relationship. In early biomaterials science, wettability was the most readily available measurement technique, with the most specific measurement of surface chemistry, achieving a 1–2nm sensing depth for the price of a simple contact angle goniometer.^9^ With the Ramé-Hart contact angle goniometer (and similar), an ever present piece of affordable equipment for measuring the sessile drop contact angle in most biomaterial laboratories, it formed the basis of surface chemical characterization in virtually all papers on the subject.

In the late 70s, the surface chemical characterization equipment developed for the semiconductor and chemical industries started to become available in biomaterial laboratories, including x-ray photoelectron spectroscopy^10^ and static secondary ion mass spectrometry (SIMS),^11^ which in tandem still provide today the most thorough characterization of chemistry for applied surfaces. For those wishing to investigate wetting phenomena more deeply, multiple liquids could be used in combination with a selection of theoretical treatments to determine the polar and van de Waals contributions to surface energy. The dynamic contact angle could be exploited to exert more control over the dynamics of the contact line utilizing a Wilhelmy plate configuration^12^ although essentially probing the same phenomena, but with the advent of the concept of super hydrophobicity, rolling drop and more complex measurement experiments were required.^13^ More recently piezodispensed picoliter liquid droplets with a footprint diameter of 50–100 *μm* have been used to measure contact angles on 300 *μ*m diameter microarray polymer spots.^14^ Unfortunately, high throughput measurement used in the microarray work cited here does not allow for the receding measurement to be made, and so, the comparison and therefore discussion will be limited to the utility of this static sessile drop WCA measurement.

We will outline some of the successes achieved in correlating water contact angle measurements with biological responses in Secs. IV and V.

## IV Protein Adsorption and Mammalian Cell Attachment To Materials

Much work has been carried out on mammalian cell attachment to materials, and so, we briefly consider water contact angle observations in this area before proceeding to consider microbial cell–surface interactions. Eukaryotic cell–material studies rationalize not only the cellular response to material chemistry in terms of the surface dictating protein adsorption from the medium in terms of the type and amount but also importantly the difficulty in measuring and therefore often experimentally overlooked aspect of the conformation of surface proteins in the excellent work by Latour.^15^ See Vogler *et al.*^5^ for an excellent recent comprehensive review on protein adsorption to materials.^5^

**Fig. 1:**
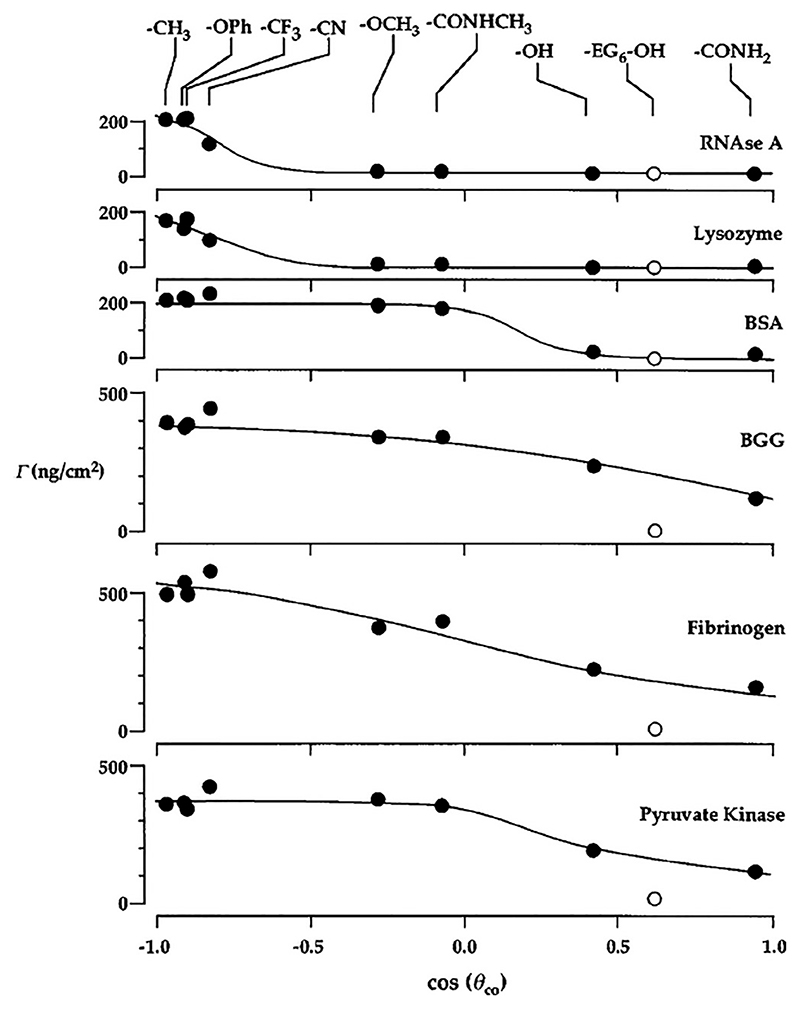
Surface density of seven adsorbed protein films on a variety of SAMs on gold as a function of the contact angle of water under cyclooctane on the as-prepared SAMs. The adsorption of solutions containing the proteins at a concentration of 1.0 mg/ml in PBS was measured by SPR. The graphs show the surface density measurement after allowing the binding reactions to proceed to completion (20min). EG6OH is indicated by an open circle to emphasize its anomalous behavior. Reprinted (adapted) with permission from Sigal *et al.,* J. Am. Chem. Soc. **120**, 3464 (1998). Copyright 1998 American Chemical Society.

Simple correlations of wettability with protein attachment have been noted when small libraries of related chemistries are employed. The study by Sigal *et al.* in 1998 on nonspecific protein adsorption to nine different self-assembled monolayer (SAM) chemistries on gold substrates is an interesting example.^16^ The authors used single component SAMS and noted monotonic trends in protein attachment, with more protein adsorbed on the less wettable surfaces measured using the water contact angle under cyclooctane (Fig. 1).

**Fig. 2:**
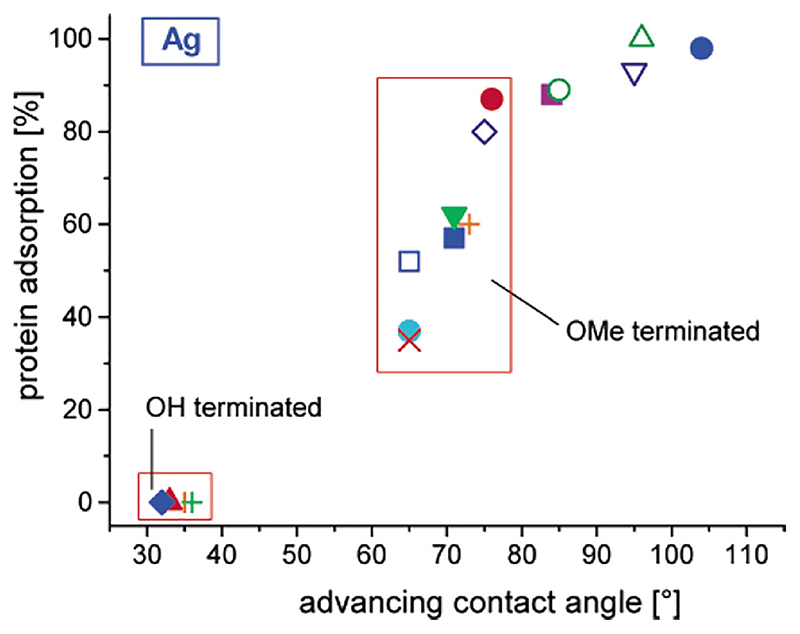
Amount of protein adsorbed on a given oligoether SAM on silver normalized to the amount of protein adsorbed on a monolayer of hexadecanethiol on gold (100%) vs advancing aqueous contact angle of the SAM. Reprinted (adapted) with permission from Herrwerth *et al.*, J. Am. Chem. Soc. **125**, 9359 (2003). Copyright 2003 American Chemical Society.

Despite the general trend of increasing protein adsorption with decreasing water wettability indicating that it may be a good general indicator of the propensity of a surface to adsorb proteins, the authors stressed that it is also necessary to consider specific structural features, for example, group dipole moment for -CN, hydrogen bonding for -CONH_2_ and CONHCH_3_, and conformational disorder for -EG_6_OHs of each surface (where EG = ethylene glycol). Further empirical work supported by molecular simulations by Herrwerth *et al*. took into consideration the structure of oligo ethylene glycol (OEG) on different metals (Fig. 2). This resulted in further classification of internal and external hydrophilicity of tethered self-SAMs of OEG as contributors to the protein resistance of the surface due to the need to coordinate water in the interior and at the surface of the monolayers to achieve optimal protein resistance.^17^ In the case of OEG SAMs, a strong theoretical basis has emerged to link protein resistance to the SAM structure involving the composition and the density (modulated by the choice of the metal on which the SAM is formed). The authors conclude that the protein resistance of OEG-thiol SAMs on gold is controlled by two primary structural features: the terminal hydrophilicity of the head group combined with the formation of a dense but disordered OEG brush with significant penetration of water into the OEG-SH SAMs. Other methods such as solvent control have been used to modulate the SAM structure and protein adsorption.^18^

In an extension of the Whitesides’ SAM work with proteins, cell attachment to libraries of SAMs on gold after protein exposure was investigated. No trends, such as seen in Fig. 1 for the adsorbed protein amount against surface wettability, were reported for cellular attachment. Indeed, low bovine capillary endothelial cell (BCE) levels on surfaces with high protein attachment were attributed to conformational differences.^19^ When the number of components was reduced from eight SAM molecules in the previous study to 2, a number of model material systems have succeeded in revealing simple WCA–cell attachment correlations. One method used to modulate the water contact angle is to form a surface chemical gradient between two components. Oxidation of polyethylene using a diffusion barrier, where one component is a virgin polymer and the second is an oxidized polymer resulted in a monotonic WCA gradient. Chinese hamster ovary, fibroblast, and endothelial cell attachment was quantified, and this revealed that a maximum cell number was observed on the gradients between 50° and 60° for all three cell types.^20^

**Fig. 3:**
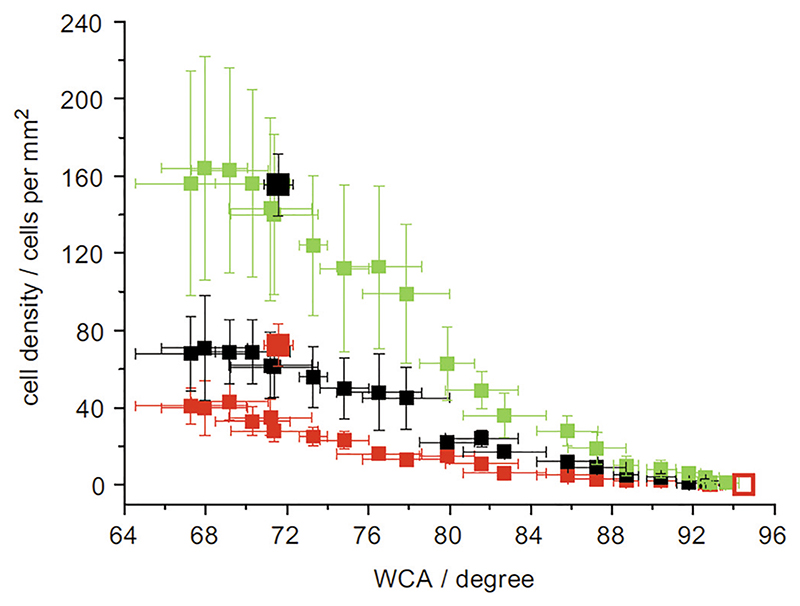
Cell number on the shallow gradient after days 1 (red), 2 (black), and 3 (green) plotted against the corresponding WCA. The uniform samples (larger symbols) are shown for day 1 (ppHex: red) and day 2 (ppHex: black). The error bars represent the standard error of the mean (gradient: n = 15; uniform samples, n = 35) (Ref. 21).

Using allylamine and hexane plasma polymer precursors to form a polymer wettability gradient from one material type to another across a surface revealed that fibroblasts adhered and proliferated preferentially on the more hydrophilic amine surfaces (Fig. 3).^21^ This shows that strong correlations between different surface energies only occur when on the *two component* surfaces, suggesting that it may be that while sometimes the correlation has been rationalized in terms of wettability, it is actually the identity of the individual surface components, which has the controlling effect on the cell response. For example, the higher fibroblast cell attachment at a lower water contact angle on allylaminehexane surfaces indicates the preference of the cells to attach to the amine chemistry of the plasma polymerized allyl amine compared to plasma polymerized hexane, rather than lower water contact angle surfaces in general.

Another report of fibroblast adhesion to a variety of glass surfaces including oxidized thiols, quaternized amines and amines, thiols, and methyl terminated surfaces showed that the WCA = 22° quaternized amine had greater cell attachment and observed enhanced cell spreading on the hydrophilic surfaces in the study relative to hydrophobic surfaces.^22^ More recently, in polymer array experiments designed specifically to include as great a diversity as possible in order to survey a large chemical space, no correlation was found between the attachment of stem cells and the water contact angle across more than 140 polymers.^23^ In the field of osteoblast–biomaterial interactions, Gentleman noted that simple correlations have been found between contact angle derived surface energy measurements in some instances, whereas in others, surface energy has not been found to control the cellular response.^4^ Thus, it is clear that there is not a simple water contact angle prediction for a given cell type within studies where diverse material libraries are considered.

Most of the materials that are well known to *resist* protein adsorption and some that resist mammalian cell attachment (when presented in the right form such as packing density, etc.) are hydrophilic, including SAMs of EG,^17^ polymer brushes of EG (Ref. 24), synthetic hydrogels such as poly(hydroxyethyl methacrylate),^25^ natural hydrogels such as alginates,^3^ and zwitterionic materials.^26^ These all have low water contact angles, if one is measurable at all, and all resist protein and cell attachment to some degree in environments of stringency ranging from short-term incubation in serum containing media to long periods in whole blood. There seems to be a strong and clear body of evidence that resistance (of proteins and mammalian cell attachment) correlates with water wettable surfaces. However, this does not seem to translate to a more general relationship with the water contact angle. Furthermore, when diverse rather^27,28^ then chemically similar libraries of materials^19,21^ are studied, it is clear that there is rarely a clear trend in the cell number versus water contact angle data.

It appears that the *devil is in the detail,* i.e., the surface chemistry, which WCA may follow, appears to be the controlling determinant for protein adsorption (including not just amount but conformation) and therefore cell adhesion. The conclusion therefore is that the WCA cannot be used as a general predictor of protein or mammalian cell attachment to surfaces.

## V Attachment of Microorganisms To Surfaces

### A Correlation of protein, bacterial, and marine spore attachment resistance

**Fig. 4:**
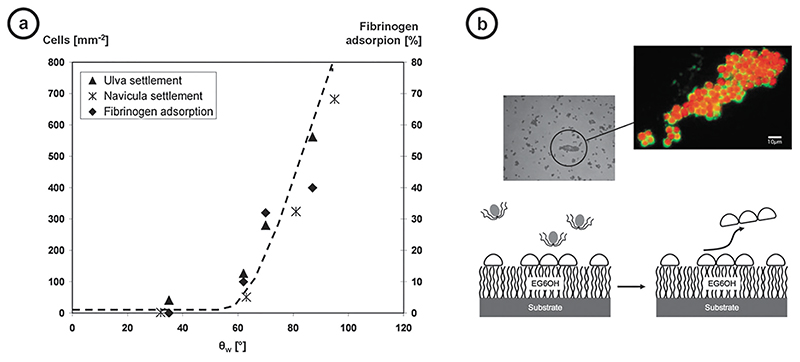
Examples of the relationship between wetting, protein resistance, and the attachment of two species of fouling algae (*Ulva linza* zoospores and cells of the diatom *Navicula* perminuta). (a) Fibrinogen adsorption and settlement of *Ulva* zoospores and cells of Navicula on a series of oligo(ethylene glycol) terminated self-assembled monolayers. (b) Schematic of raft removal and microscopy images of rafts of *Ulva* spores on and removed from the surface of a dish containing an EG_6_OH-terminated alkanethiol SAM. Spores in the fluorescence image are autofluorescent, and the spore adhesive has been stained with mABEnt6. Scale bar *¼* 10 *μ*m. Reproduced (in part) with permission from Rosenhahn *et al.,* Phys. Chem. Chem. Phys. 12, 4275 (2010). Copyright 2010 The Royal Society of Chemistry.

Materials with resistance to bacterial attachment have sometimes been held to be synonymous with materials with resistance to protein adsorption. This view has been specifically explored and supported in a study on polymers^29^ and model SAM studies19 from the Whitesides’ group. They used six SAMs, identifying a weak correlation between BCE cell resistance and that of microbes. They also identified some surfaces that matched and even surpassed EG in resisting adhesion of *Staphylococcus aureus* and *Staphylococcus epidermidis* and attachment and spreading of BCE cells. A strong relationship has been observed between the water contact angle measured on a range of hydroxyl-, methoxyl-, ethoxyl-, propoxyl-terminated EG hexamers on gold and adsorbed fibronectin and settled marine fouling algae spores (Fig. 4).^2,30^

### B Recent evidence to support WCA as a poor predictor of the bacterial response to diverse polymer libraries

Looking at *Pseudomonas aeruginosa* attachment to and biofilm formation on a wide library of polymers presented in a microarray format, Sanni *et al.* found that there was no relationship with WCA in the narrow range of materials considered which constituted those found to resist biofilm formation (in the range of 80°–90°).^31^ They noted instead that parameters related to the hydrophobicity (*clog P*) and molecular flexibility (number of rotatable bonds = *nRoTB)* when combined in the alpha parameter (α = *0.44nRoTB—c logP*) strongly correlated with resistance to attachment and subsequent biofilm development. However, the caveat to this observation is that this strong relationship was identified only for a subset of 21 attachment resistant homopolymers from the total library of 140 monomers. Expanding the library beyond these (meth)acrylates with pendant hydrocarbons prevented any simple trends being identified. In contrast, models where all the materials have been identified using sparse feature selection of large molecular descriptor libraries using neural networks did identify correlations. These did however require more arcane molecular descriptors to predict bacterial attachment but do offer hope that this approach can identify a framework to describe biofilm formation in terms of the material structure.^32^

Most recently, in an expansion of the Epa *et al*. work, we have used a multilinear regression model to compare the ability of WCA, ToF SIMS ions and molecular descriptors to describe the attachment and biofilm formation of *P. aeruginosa*, *Staphylococcus aureus*, and *Escherichia coli* across a large and diverse polymer library in minimal media designed to stress the bacteria into forming biofilms.^33^ In the multipathogen model, WCA had an insignificant contribution to prediction, in comparison with alternative methods of using secondary ion intensities from ToF SIMS or molecular descriptors. This points to the importance of the richness of structural information in the mass spectrometry and the quantitative structural descriptors in describing the surface chemistry, which are clearly controlling factors for biofilm formation in these large diverse polymer libraries under the culture conditions employed.

### C Cells treated as inert particles that will attach to a surface, or not, based upon physicochemical rules

As a logical “sanity check,” let’s ask....*why would a number relating to the wetting of a surface be expected to correlate with cellular attachment across diverse materials?* If cells were controlled simply by physicochemical laws, we could think of their attachment as a consequence of a simple interaction between two surfaces of different energies. This could provide a strong relationship with WCA. The Derjaguin, Verwey, Landau and Overbeek theory explains the quantitative aggregation of aqueous dispersions and describes the force between charged surfaces interacting through a liquid medium. It has been applied to bacterial cells using theories that assume that microbial cells behave as inert particles. However, although this reductionist approach is attractive to many, most cells, both eukaryotic and prokaryotic, respond on contact with a surface in a dynamic way, making decisions as to whether or not to attach rather than behaving as inert particles. For example, the genome of *Pseudomonas aeruginosa* contains ~6000 genes, around 10% of which are devoted to environmental sensing and adaptation.^34^ These include over 60 sensor regulator pairs, riboregulators, and second messengers environmental offer such as cyclic-diguanylate, which contribute to sophisticated gene regulatory networks capable of receiving and integrating multiple external signals to determine the downstream bacterial behavioral changes.^35^ A number of these signal transduction pathways are involved in surface sensing via surface appendages such as flagella, pili, and membrane associated sensor proteins.

Type IV pili and flagella enable *P. aeruginosa* to move as single cells or as communities over surfaces via swimming, swarming, or twitching motility as well as acting as surface probes to help the bacteria decide whether to switch from a motile to a sessile lifestyle and form a biofilm.^36^ With respect to the latter, extracellular polymeric substances are produced by attached bacterial cells, guiding movement in the case of biofilm microcolony formation^37^ and inevitably influencing surface adhesion strength. Given that bacteria are capable of such sophisticated surface sensing (as are mammalian cells), it should not be much of a surprise that WCA alone is an insufficient predictor of bacterial attachment. Such complex cellular recognition phenomena clearly have the likelihood of acting as a necessary factor in the prediction of cellular attachment to surfaces and confound any simple physicochemical interpretation where surface energetics and WCA may play a role. There is however the possibility that the relative magnitude of the physicochemical adhesion effects can be dominant in some environmental circumstances, compared to the biological recognition phenomena—a case presented by Rouxhet *et al*. for a range of materials.^38^ On the basis of surface energetic measurements complemented by surface chemical and zeta potential analysis, they concluded for a range of metal, plastic, and glass surfaces that bacterial and fungal surface charge and energy were the determining factors in cell adhesion in their experiments. They carried out the experiments using careful sedimentation protocols to minimize any hydrodynamic effects and concluded that they could predict attachment under these very controlled conditions based on thermodynamic interfacial surface energy and electrostatic interactions for a range of *p*Hs. This contrasts with most experiments, which are carried out at a single *pH*, normally involving intentional washing steps, flow during culture for aeration purposes, or even unintentional flow upon removal from culture. In our view, such a flow challenge of the cell–surface adhesion is critical in modeling the *in vivo* environment with *in vitro* testing and represents possibly the most important variable between different testing protocols that may influence bacterial attachment and biofilm formation in work from different laboratories.

It is highly likely that there is no universal explanation for the attachment of different bacterial species across different surfaces although tantalizing glimpses of correlations between material chemistry descriptions and resistance to biofilm formation of multiple micro-organisms are emerging.^32,33^ To convert these exciting findings in the almost infinite biomaterial chemical space into a working hypothesis for improving biomaterials, the fundamentals of attachment need to be elucidated for both individual organisms and microbial communities. This will require full characterization of in *vitro* systems in terms of both surface chemistry and intra- and interbacterial surface sensing signal transduction mechanisms and careful control of bacterial culture and any flow challenge conditions to which the cell–surface interface is subjected.

In summary, we have outlined our thoughts on the question of using wettability or the water contact angle in predicting mammalian and microbial cellular responses, indicating the poor predictive properties when dealing with diverse libraries of material surface chemistry. We have highlighted a series of relatively hydrophobic polymers that resist bacterial attachment and biofilm formation to a range of pathogens. Through this observation, we conclude that these act by a mechanism that remains to be elucidated and is distinct from that involving interfacial water association at the surface, which is invoked for ethylene glycols. We note that molecular descriptors have been found to correlate with bacterial attachment but as yet without the proposition of a causative mechanism. We anticipate a significant role of bacterial surface sensing in these observations and highlight how *in vitro* culture conditions differ throughout the literature, in particular how media flow or postculture washing steps can be critical in disrupting the adhesion of bacterial cells to material surfaces.

## References

[1] Castner D, Ratner B (2002). Surf Sci.

[2] Rosenhahn A, Schilp S, Kreuzer HJ, Grunze M (2010). Phys Chem Chem Phys.

[3] Banerjee I, Pangule RC, Kane RS (2011). Adv Mater.

[4] Gentleman MM, Gentleman E (2014). Int Mater Rev.

[5] Vogler EA (2012). Biomaterials.

[6] Engler AJ, Sen S, Sweeney HL, Discher DE (2006). Cell.

[7] Dalby MJ, Gadegaard N, Tare R, Andar A, Riehle MO, Herzyk P, Wilkinson CDW, Oreffo ROC (2007). Nat Mater.

[8] Schumacher JF, Carman ML, Estes TG, Feinberg AW, Wilson LH, Callow ME, Callow JA, Finlay JA, Brennan AB (2007). Biofouling.

[9] Bain CD, Whitesides GM (1988). J Am Chem Soc.

[10] Ratner B, Weathersby PK, Hoffman AS, Kelly MS, Sharpen LH (1978). J Polym Sci.

[11] Castner DG, Ratner BD, Grainger DW, Kim SW, Okano T, Suzuki K, Briggs D, Nakahama S (1992). J Biomater Sci Polym Ed.

[12] Vogler EA (1998). Adv Colloid Interface Sci.

[13] Li XM, Reinhoudt D, Crego-Calama M (2007). Chem Soc Rev.

[14] Taylor M, Urquhart AJ, Zelzer M, Davies MC, Alexander MR (2007). Langmuir.

[15] Sivaraman B, Latour RA (2010). Biomaterials.

[16] Sigal GB, Mrksich M, Whitesides GM (1998). J Am Chem Soc.

[17] Herrwerth S, Eck W, Reinhardt S, Grunze M (2003). J Am Chem Soc.

[18] Li L, Chen S, Zheng J, Ratner BD, Jiang S (2005). J Phys Chem, B.

[19] Ostuni E, Chapman RG, Liang MN, Meluleni G, Pier G, Ingber DE, Whitesides GM (2001). Langmuir.

[20] Lee JH, Khang G, Lee JW, Lee HB (1998). J Colloid Interface Sci.

[21] Zelzer M, Majani R, Bradley JW, Rose FRAJ, Davies MC, Alexander MR (2008). Biomaterials.

[22] Webb K, Hlady V, Tresco PA (1998). J Biomed Mater Res.

[23] Celiz AD (2014). Biomater Sci.

[24] Hucknall A, Rangarajan S, Chilkoti A (2009). Adv Mater.

[25] Chirila TV, Constable IJ, Crawford GJ, Vijayasekaran S, Thompson DE, Chen Y-C, Fletcher WA, Griffin BJ (1993). Biomaterials.

[26] Cheng G, Li G, Xue H, Chen S, Bryers JD, Jiang S (2009). Biomaterials.

[27] Mei Y (2010). Nat Mater.

[28] Hook AL (2012). Nat Biotechnol.

[29] Chapman RG, Ostuni E, Liang MN, Meluleni G, Kim E, Yan L, Pier G, Warren HS, Whitesides GM (2001). Langmuir.

[30] Schilp S, Kueller A, Rosenhahn A, Grunze M, Pettitt ME, Callow ME, Callow JA (2007). Biointerphases.

[31] Sanni O, Chang C-Y, Anderson DG, Langer R, Davies MC, Williams PM, Williams P, Alexander MR, Hook AL (2015). Adv Healthcare Mater.

[32] Epa VC (2014). Adv Funct Mater.

[33] Mikulskis P Broad-spectrum mathematical models of pathogen attachment to materials.

[34] Stover CK (2000). Nature.

[35] O’Toole GA, Wong GCL (2016). Curr Opin Microbiol.

[36] Kazmierczak BI, Schniederberend M, Jain R (2015). Curr Opin Microbiol.

[37] Zhao K, Tseng BS, Beckerman B, Gibiansky ML, Harrison JJ, Luijten E, Parsek MR, Wong GCL (2013). Nature.

[38] Mozes N, Marchal F, Hermesse MP, Van Haecht JL, Reuliaux L, Leonard AJ, Rouxhet PG (1987). Biotechnol Bioeng.

[39] Stevens MM, George JH (2005). Science.

[40] Ratner BD (2007). Biomaterials.

[41] Celiz AD (2014). Nat Mater.

